# Proteomic Studies in Absence Epilepsy: A Systematic Review of Methodological Diversity and Implications for Data Interpretation

**DOI:** 10.3390/cimb48020200

**Published:** 2026-02-11

**Authors:** Aslihan Gunel

**Affiliations:** 1 Institute of Health Sciences, Department of Neuroscience, Acibadem Mehmet Ali Aydinlar University, 34638 Istanbul, Türkiye; aslihan.gunel@live.acibadem.edu.tr or agunel@ahievran.edu.tr; Tel.: +90-(386)-280-4524; 2 Department of Chemistry-Biochemistry, Faculty of Science and Arts, Kırsehir Ahi Evran University, 40200 Kirsehir, Türkiye; 3 Department of Molecular Biology and Genetics, Faculty of Science and Arts, Kırsehir Ahi Evran University, 40200 Kirsehir, Türkiye

**Keywords:** absence epilepsy, proteomics, GAERS, WAG/Rij, sample preparation

## Abstract

Absence epilepsy (AE) is a common pediatric epilepsy syndrome marked by brief lapses in consciousness and characteristic 2.5–4 Hz spike-and-wave discharges on EEG. Although its clinical and electrophysiological features are well established, the molecular mechanisms underlying AE remain incompletely understood. Proteomic approaches offer a powerful means to explore these mechanisms; however, their application in AE remains limited and methodologically heterogeneous, which complicates data integration. In this review, proteomic methodologies applied in rodent models of absence epilepsy are critically examined, including genetic rat models such as Genetic Absence Epilepsy Rats from Strasbourg (GAERS) and Wistar Albino Glaxo rats from Rijswijk (WAG/Rij), monogenic mutant mouse models, and pharmacologically induced models. The technical workflow is described particularly, from tissue sampling and protein preparation (including gel-based and gel-free methods) to mass spectrometric analysis using data-dependent and data-independent acquisition strategies. Emerging technologies such as spatial proteomics, Trapped Ion Mobility Spectrometry coupled with Parallel Accumulation–Serial Fragmentation (TIMS-PASEF), and the integration of artificial intelligence are also evaluated in relation to their potential to address current technical limitations. Beyond synthesizing convergent molecular pathways including synaptic dysfunction, altered energy metabolism, and neuroinflammation, the review examines how methodological choices—such as model selection, brain region dissection, sample preparation protocols, and analytical platforms—contribute to experimental outcomes and data interpretation. By integrating current evidence with a focus on methodological aspects, this review provides a framework for designing more robust, reproducible, and clinically relevant proteomic studies in absence epilepsy.

## 1. Introduction

According to the ILAE 2017 [1] classification, absence epilepsy is classified as a generalized epilepsy syndrome with a strong genetic component. Absence epilepsy is characterized by brief episodes of unconsciousness lasting 3–30 s, accompanied by spike-and-slow wave discharges at 2.5–4 Hz [2,3]. Three main theories have been proposed to explain the pathophysiology of absence epilepsy. The first, the “centrencephalic” theory, proposes that epileptic discharges arise from a deeply situated, diffuse subcortical pacemaker within the thalamus, a concept that was later reinterpreted in 1991 as originating from the thalamic clock. The second, the “corticoreticular” theory (1968), posits that rhythmic thalamic spindle oscillations transform into spike-wave discharges when excessively driven by cortical input. Finally, the “cortical focus” theory is based on findings from animal experiments. According to the cortical focus theory, seizure initiation occurs in the cortex, which subsequently engages the thalamus, leading to a reciprocal cortico-thalamic interaction that amplifies rhythmic discharges [4].

The first genetic alteration identified in human absence epilepsy was the arginine-to-glutamine substitution at position 43 (R43Q) in the gamma-aminobutyric acid type A (GABA_A_) receptor [5]. While a causal relationship between mutations in GABA_A_ receptor subunits and absence epilepsy has been established, such mutations are not observed across all forms of absence epilepsies. Several families with childhood absence epilepsy also have mutations in other channel proteins besides GABA receptors. These include calcium and potassium channel proteins, with particular emphasis on T-type Ca^2+^ channel proteins [6,7].

Following the completion of the Human Genome Project, there was an expectation that curative strategies for epilepsies with known genetic causes could be identified. However, no cure for epilepsy has been identified to date, a limitation that may reflect the fact that these disorders are not necessarily driven by a single genetic mutation. In addition, the same mutation may give rise to multiple conditions, while distinct molecular pathways can be affected by different genetic alterations. Deciphering the proteomes of affected tissues is therefore crucial to uncover the molecular processes underlying these complex mechanisms. For example, recent studies on post-traumatic epilepsy demonstrate the utility of proteomic approaches in identifying a novel therapeutic target [8].

Epilepsy affects not only patients themselves but also has a substantial impact on the lives of their families and caregivers. Understanding the underlying molecular mechanisms and identifying effective therapeutic targets and treatment strategies are necessary to reduce this burden. However, since obtaining human brain tissue or cerebrospinal fluid (CSF) is invasive and practically difficult in absence epilepsy, research relies mainly on experimental animal models. Consequently, a major challenge in interpreting and integrating proteomic data in absence epilepsy is the significant methodological heterogeneity across existing studies, encompassing choices of animal models, sample preparation protocols, and analytical platforms. Integrating various proteomic methods (gel-based and shotgun) is crucial to fully understand the complex mechanisms of absence epilepsy. In contrast to previously published reviews that broadly addressed proteomic approaches in epilepsy, the present review places specific emphasis on the methodological aspects of absence epilepsy proteomics rather than solely summarizing molecular outcomes. Accordingly, differences in experimental design and analytical workflows are examined in relation to their impact on reproducibility and data interpretation, allowing this review to be distinguished from the broader literature. This review synthesizes molecular findings from proteomic studies in rodent models of absence epilepsy while critically examining the methodologies employed. By critically appraising the technical landscape, the review highlights sources of variability across studies and outlines considerations that may support improved reproducibility and clinical relevance in future proteomic research. There is a growing need for proteomic studies that are both cell-type-specific and focused on post-translational modifications in active neural circuits, with attention to the distinct characteristics of different epilepsy subtypes. This is particularly important because the mechanisms underlying epilepsy are still not fully understood, and achieving a complete treatment requires going beyond symptom control to addressing the disease itself after its onset. To provide a clearer framework for the subsequent sections, the proteomic studies included in this review are summarized in Table 1.

## 2. Materials and Methods

**Figure 1:**
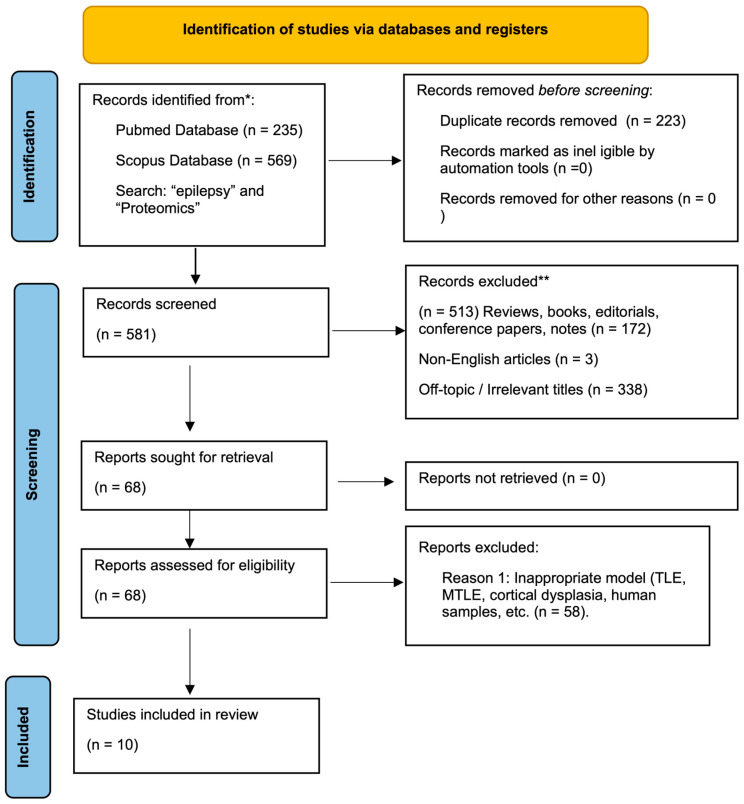
PRISMA 2020 flow diagram illustrating the systematic literature search and selection process. The search was conducted across PubMed and Scopus databases covering the period from 2002 to 2025. The diagram details the number of records identified, screened for duplicates and relevance, and excluded based on specific criteria (e.g., inappropriate animal models, non-proteomic methods), resulting in the final selection of studies included in this review. * Records identified through database searching (PubMed and Scopus). ** Records excluded after title and abstract screening; reasons for exclusion are listed in the figure.

A comprehensive search was performed using two widely used databases, PubMed and Scopus, covering studies published between 2002 and 2025. The search was performed using the following keyword combinations: ‘absence epilepsy’ AND ‘proteomics’, and ‘epilepsy’ AND ‘proteomics’. Articles were screened for relevance based on titles and abstracts. Full-text manuscripts were selected according to the following criteria: (1) original research articles employing proteomic technologies; (2) studies conducted in established genetic (e.g., GAERS, WAG/Rij, stargazer) or pharmacological (e.g., GBL-induced) rodent models of absence epilepsy. The Prisma workflow is presented in Figure 1. The PRISMA 2020 checklist is provided as Supplementary Materials.

The selected studies were systematically analyzed with a primary focus on their methodological design. Data were extracted and compared on the following: (1) the animal model utilized; (2) brain region(s) dissected; (3) sample preparation protocols (e.g., lysis buffers, subcellular fractionation); (4) protein separation and quantification techniques (e.g., 2-DE, 2D-DIGE, LC-MS/MS, DIA); and (5) key proteomic findings. Given the limited number of studies and their significant methodological heterogeneity, a formal meta-analysis was not feasible.

Accordingly, a qualitative and critical synthesis was conducted, with a focus on the technical and analytical challenges within the field and on how methodological choices influence data interpretation and reproducibility

## 3. Results and Discussion

### 3.1. Findings from Proteomic Studies in Epilepsy

#### 3.1.1. General Proteomic Studies in Epilepsy

Since the second half of the 1990s [18], when the term proteomics first appeared in the literature, studies on epilepsy and epileptogenesis have been conducted using various rodent models that mimic the epileptic and epileptogenic processes in human tissues, as well as in humans themselves. Zebrafish (*Danio rerio*) epilepsy models have been introduced to animal models in recent years due to their high genetic similarity to the human genome, expressing around 84% of the genes known to be connected in human diseases, and the relatively low cost of experimental procedures [19,20].

Upon reviewing the proteomic studies conducted on neurological disorders, it is observed that the predominant samples utilized comprise either human post mortem specimens and/or cerebral sections procured subsequent to surgical intervention, or animal models that accurately emulate the disease. Another important aspect is which proteomic approaches are used in these studies. The utilization of 2D-gel electrophoresis and diverse shotgun proteomic methodologies, which were initially employed during the inception of proteomics, have been observed in research pertaining to epilepsy. The most extensively studied epilepsies are temporal lobe epilepsy (TLE), which is categorized as focal epilepsies, and focal cortical dysplasia, characterized by cortical malformations. The literature on epilepsy is highly heterogeneous and complex with affected brain regions and pathways. The studies conducted up to 2020 that examined these diseases from a variety of perspectives, as well as their findings, are compiled in [21], and the studies conducted since 2014 are compiled in [22]. This existing heterogeneity in methodologies and focus areas underscores the necessity for the present review, which aims to provide a dedicated critical analysis specifically for absence epilepsy models, with an emphasis on the technical approaches that have been employed.

#### 3.1.2. Proteomics in Absence Epilepsy Models

In recent years, proteomics has emerged as a valuable approach to uncover molecular mechanisms underlying absence epilepsy. This section provides an overview of the animal models used in such studies and summarizes the existing literature on this topic. Most frequently used animal models in proteomic research on absence epilepsy are GAERS and WAG/Rij models. These are well-validated genetic models of typical absence epilepsy [23,24]. When comparing the EEG phenotypes of GAERS and WAG/Rij rats, scientists found that the former exhibit a greater frequency, cumulative duration, and mean duration of spike-wave discharges (SWD). SWDs in WAG/Rij rats tend to occur more frequently during cycles in the WAG/Rij rats [25]. Differences in disease onset and progression between GAERS and WAG/Rij rats may influence the timing of tissue collection and contribute to variability among proteomic datasets. Animal models of absence epilepsy include not only this polygenic spontaneous models [26] but also monogenic mouse models like Tottering and Lethargic Ducky, and pharmacologically induced models, such as the gamma-butyrolactone (GBL) model [16]. While the inclusion of chemically induced models like GBL provides insights into acute seizure mechanisms, it is important to note the interpretative challenges when comparing their proteomic profiles with those of chronic genetic models, as the pathophysiology and time course of proteomic alterations may differ significantly.

This review summarizes the proteomics research and how proteomic methods were applied in studies on absence epilepsy. A critical comparison of the methodologies employed across these studies reveals significant diversity in sample preparation, separation techniques, and mass spectrometric analysis, which must be considered when integrating their findings.

##### Genetic Models of Absence Epilepsy

The conducted proteomic studies on genetic models of absence epilepsy and their results are summarized below.

Proteomic studies in genetic mouse models of absence epilepsy have primarily employed gel-based approaches. The study by Ryu et al. [15] was performed in the stargazer mutant mouse model; the thalamus was investigated mainly, but also some proteins were changed in the same manner in both the cortex and hippocampus. The proteins detected in the study were categorized as metabolic disease, oxidative stress response, nerve system development, and neurological disease. Most identified proteins were down-regulated in the stargazer mutant mice, with the exception of the acidic form of peroxiredoxin 6 (PRX6) and mortalin.

The proteomic study was performed by the GAERS model by 2-DE by [9,10]. The study of Danış et al. [9] encompasses the parietal cortex, thalamus, and hippocampus of the animals, and the study of Yuce-Dursun et al. [10] examined the cortex of the GAERS. The results of the study by Danış et al. [9] obtained myelin basic protein and macrophage migration inhibitory factor in the thalamus, O-beta 2-globulin and macrophage migration inhibitory factor in the hippocampus, and 14-3-3 ς and ATP synthase subunit delta in the parietal cortex; the method applied in this study was 2-DE combined nano LC-MS/MS. In the study by Yuce-Dursun et al. [10], the differentiated proteome of the membrane fraction of cortex tissue used 2-DE coupled with matrix-assisted laser desorption/ionization mass spectroscopy (MALDI). 14-3-3η was increased and guanine nucleotide-binding protein G(I)/G(S)/G(T) subunit beta-1 was significantly down-regulated in GAERS.

A notable shotgun proteomic study was conducted in the GAERS rat model by Harutyunyan et al. [11], as part of a multi-omics investigation. Shotgun proteomics first introduced by Yates et al. [27] involved the digestion of proteins into peptides, which were then separated and analyzed using tandem mass spectrometry (MS/MS). Applications of shotgun proteomics in absence epilepsy research remain limited to Harutyunyan et al. [11] up to date. This study integrated metabolomic and proteomic analyses of the somatosensory cortex and thalamus; 102 and 123 proteins were benchmarked in the indicated region respectively. Upregulated pathways included the synthesis, transport, and clearance of neurotransmitters, synaptic signaling, and oxidative processes. On the other hand, the down-regulated pathways were associated with lysine catabolism, the GTPase cycle, the breakdown of galactose and glycogen, regulation of necrosis, and the innate immune system. This study represents the most comprehensive proteomic profiling of GAERS to date, highlighting pathway-level alterations that are not readily captured by gel-based approaches. Overall, GAERS-associated proteomic alterations predominantly involved synaptic signaling, energy metabolism, and stress-related pathways, with region-specific differences across cortical and thalamic structures.

Importantly, the multi-omics study by Harutyunyan et al. [11] also included behavioral assessments, such as the sucrose preference test, allowing molecular findings to be interpreted in a functional context. At present, this study represents the only proteomic-based investigation in absence epilepsy that directly integrates molecular profiling with behavioral readouts. This is particularly relevant because genetic models of absence epilepsy, including GAERS and WAG/Rij rats, are known to display neurobehavioral comorbidities beyond seizures, such as anxiety-like behavior, anhedonia, and cognitive alterations. These features have been consistently documented in detailed behavioral phenotyping studies, emphasizing that absence epilepsy involves broader affective and cognitive disturbances in addition to spike-and-wave discharges [28]. In this regard, linking proteomic alterations with behavioral outcomes represents an important step toward understanding the functional significance of molecular changes identified in animal models of absence epilepsy.

Another widely used rat model of absence epilepsy is the WAG/Rij strain. Notably, there have been three proteomic studies conducted using this particular model.

Györffy et al. [12] examined the effects of lipopolysaccharide (LPS) on seizure patterns in the WAG/Rij rat model, with a focus on the parietal cortex and thalamus. The results demonstrated that LPS exposure altered seizure patterns in the WAG/Rij model. Proteomic analysis revealed altered protein expression in both the parietal cortex and hippocampus, affecting 15 proteins in the former and 28 proteins in the latter. In this study, the 2-DIGE-LC-MS/MS method was employed to investigate the proteome alterations. Protein network modeling indicated that the observed proteomic changes were predominantly associated with immune response activation, including involvement of the NFκB signaling pathway.

Gürol et al. [13] examined proteomic alterations in two rat age groups, namely 2-month-old and 6–8-month-old animals. These age groups were selected because rats typically develop mature seizures after approximately four months of age. The methodology employed in this study involved the utilization of a 2-DE technique in conjunction with matrix-assisted laser desorption/ionization time-of-flight mass spectrometry (MALDI-TOF/MS). In the frontal cortex, proteins associated with vesicular transport, cell signaling, cell structure, and serine biosynthesis were identified. In contrast, the thalamus showed emergence of proteins related to cytoskeletal organization, neurite growth, and the modulation of protein and steroid synthesis. The third proteomic study in the WAG/Rij model was conducted by Sahin et al. [14] and 2-DE combined MALDI-TOF/TOF method was used in this study. In the result of the study, ERP57 decreased in both cortex and thalamus. Overall, proteomic alterations in the WAG/Rij model predominantly involved immune-related pathways, cytoskeletal organization, and metabolic processes, with age- and region-dependent differences across cortical and thalamic structures.

In addition to well-validated genetic models of absence epilepsy, proteomic studies have also been performed in genetically defined seizure-prone mouse lines that exhibit absence-like features. Lagarrigue et al. [17] conducted a research in a genetic model proposed for absence epilepsy. This study utilized BS/Orl (seizure-prone) and BR/Orl (seizure-resistant) mice. It is important to note that while the authors of the study describe these lines as a genetic model ‘in mirror’ of absence epilepsy, it is crucial to highlight that the BS/Orl mouse is primarily a model of audiogenic seizures [29], not typically characterized by the hallmark 3 Hz spike-and-wave discharges of classic absence epilepsy. Despite this discrepancy, the investigators employed MALDI imaging to compare these mirror lines and identified Synapsin-I as a potential marker distinguishing the seizure-prone and seizure-resistant phenotypes.

##### Pharmacological Models of Absence Epilepsy

The study [16] examined the alterations in the proteome caused by gamma-butyrolactone (GBL), a gamma-aminobutyric acid (GABA) analog, in the thalamus in a manner that varied with time (5, 10, 30 min). Proteomic profiling was performed using a gel-based approach based on two-dimensional differential gel electrophoresis (2-D DIGE), followed by mass spectrometric protein identification. Proteomic alterations were most prominent 10 min after GBL administration, whereas by 30 min, the proteome profile returned to baseline. The proteomic alterations of this study were grouped as cytoskeleton rearrangement, neuroprotection, neurotransmitter secretion, calcium binding, and metabolism. All identified proteins except serine protease inhibitor EIA were down-regulated. All these proteins were related to neuronal function or neurological disorders including Alzheimer’s disease, schizophrenia, and epilepsy. In particular, mouse stress inducible phosphoprotein 1 (STIP1), a co-chaperone and neuroprotector protein, was observed as destabilized during the absence seizure. The other remarkable proteins in this study were collapsin response mediator protein (CRMP) isoforms, with phosphorylation detected at S522.

Notably, several protein alterations identified in the GBL model partial overlapped with those reported in the monogenic absence epilepsy model [15], including changes in oxidative stress-related proteins and enzymes involved in energy metabolism. This convergence suggests that, despite fundamental differences in disease chronicity and etiology, the acute pharmacological and monogenic stargazer model [15] may share common downstream molecular responses associated with absence-like seizure activity.

##### Single Cell Proteomics

Single-cell RNA sequencing (scRNA-seq) has been used to investigate gene expression patterns in epilepsy [30,31,32], however, no such studies have yet been conducted specifically in absence epilepsy. The field of single-cell proteomics remains at an early stage of development [33]. Elucidating the complex mechanisms underlying disorders such as epilepsy requires investigation of cellular heterogeneity and identification of cell subpopulations with distinct protein expression profiles. When applied to the brain during epileptic episodes, single-cell proteomics enables analysis of cell-to-cell communication and the surrounding cellular milieu [34]. Neurons, glial cells, and immune cells represent major brain cell types that can be resolved using single-cell proteomic approaches. Profiling the protein composition of these cells may offer insights into their potential roles in epilepsy pathophysiology and treatment.

The application of single-cell proteomics to absence epilepsy represents a promising future direction for addressing the limitations of bulk tissue analysis, which can obscure cell-type-specific changes. Its implementation requires careful optimization of sample preparation from specific brain nuclei involved in cortico–thalamo–cortical circuits to preserve cellular integrity and protein states, reflecting an important methodological consideration.

Importantly, the rationale for applying single-cell proteomics to absence epilepsy extends beyond resolving neuronal subpopulations and includes the investigation of non-neuronal cell contributions, particularly astrocytes. Accumulating evidence indicates that astrocytes play an active role in the generation, modulation, and propagation of spike-and-wave discharges within cortico–thalamo–cortical circuits [35]. Astroglial regulation of extracellular potassium and neurotransmitter homeostasis, gap junction coupling, gliotransmission, and inflammatory signaling have been shown to critically influence network excitability and synchronization in genetic models of absence epilepsy. Notably, astrocytic dysfunction and reactive astrogliosis were reported in both GAERS and WAG/Rij rats, often preceding seizure onset, suggesting a potential causal contribution rather than a secondary response to epileptic activity [35]. In this context, single-cell proteomics offers a unique opportunity to dissect cell-type-specific protein expression patterns and signaling pathways in astrocytes and other non-neuronal populations that cannot be resolved using bulk tissue approaches. Such resolution may be particularly relevant for identifying astrocyte-driven metabolic, inflammatory, and synapse-modulatory mechanisms that contribute to absence epilepsy pathophysiology.

Current research in single-cell proteomics is primarily focused on methodological advancements, including optimization of instrumentation and sample preparation techniques for efficient protein extraction from individual cells [36]. Algorithms originally developed for bulk proteomics are also frequently applied in single-cell workflows. Accordingly, adaptation of these algorithms is necessary to maintain data reliability in single-cell proteomic analyses [37].

Despite existing limitations, recent technological advances have enabled single-cell proteomics at a limited scale. Methods such as mass cytometry (CyTOF) [38] and proximity extension assay (PEA) [39] have been adapted to investigate the proteomes of individual cells. When combined with advanced computational tools, these approaches may facilitate improved resolution of epilepsy-related processes at the single-cell level. In the context of absence epilepsy, future single-cell proteomic studies could examine proteomic differences within specific neuronal populations of the thalamus and cortex, providing finer resolution into molecular mechanisms underlying spike-wave discharge generation. Such efforts depend on the development of standardized protocols tailored to the challenges of brain tissue dissociation and low protein abundance in single cells, consistent with the broader emphasis on methodological standardization throughout this review.

##### Impact of Methodological Variables on Proteomic Data Interpretation

Comparison of the available studies shows that inconsistencies in reported proteomic findings are more often attributable to methodological differences rather than true biological contradictions. A prominent source of variability is the distinct genetic background of the experimental models. GAERS rats harbor a gain-of-function mutation in the *Cacna1h* gene (Cav3.2), whereas WAG/Rij rats exhibit a different channelopathy profile, including reduced expression of *Hcn1* and *Hcn2* in the somatosensory cortex (SSCx) and compensatory up-regulation of *Hcn4* [40,41,42]. Pharmacological models such as γ-butyrolactone (GBL) primarily reflect acute seizure-related metabolic stress and generate proteomic profiles that differ fundamentally from the progressive epileptogenesis characteristic of genetic models [43,44]. As a result, model selection directly influences the protein targets identified across studies.

Tissue sampling strategies further contribute to inter-study variability. Even when analyses focus on relevant regions such as the epileptogenic SSCx or frontoparietal cortex, the use of bulk tissue homogenates introduces a dilution effect, whereby proteomic changes occurring in specific neuronal subpopulations may be masked by abundant and relatively stable glial or vascular proteins. In addition, differences in analytical platforms influence protein detectability. Earlier studies relying on two-dimensional gel electrophoresis (2-DE) were inherently biased against hydrophobic membrane proteins, limiting the detection of ion channels that are central to absence epilepsy pathophysiology. By contrast, contemporary LC–MS/MS-based workflows offer improved coverage of these protein classes, contributing to discrepancies between earlier and more recent datasets. From a methodological perspective, several technical factors further contribute to the heterogeneity observed across proteomic datasets in absence epilepsy.

A direct comparison of the findings from these two gel-free proteomic studies is challenging due to fundamental differences in their methodological design. The study by Lagarrigue et al. [17] utilized MALDI-imaging in an acute pharmacological mouse model producing absence-like spike-wave discharges, focusing on spatial protein distribution, while Harutyunyan et al. [11] employed a bulk-tissue, multi-omics approach in GAERS rats. This disparity in models (mouse vs. rat), analytical focus (imaging vs. bulk tissue), and platforms (MALDI vs. LC-DIA-MS/MS) exemplifies the methodological heterogeneity in the field and underscores the difficulty in integrating data across studies.

**Figure 2:**
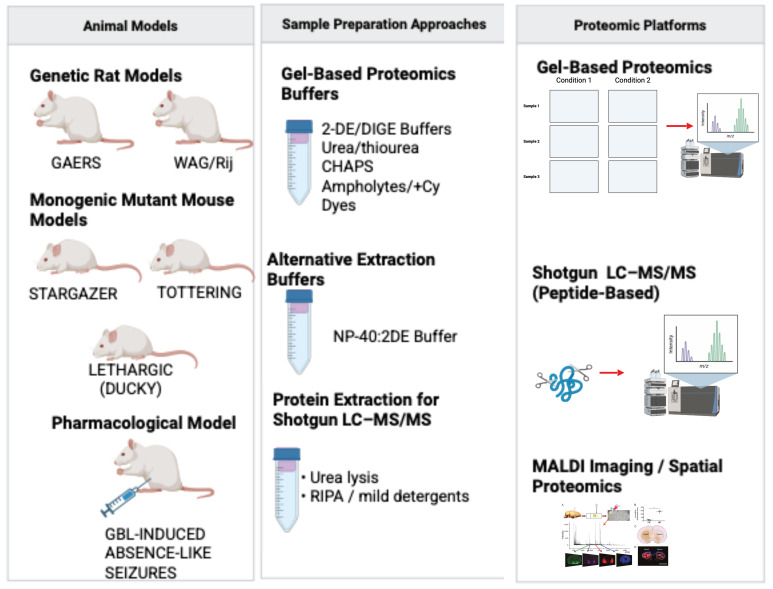
Summative overview of methodological variation in absence epilepsy proteomics: This figure summarizes major sources of heterogeneity across absence epilepsy proteomic studies, including differences in animal models (e.g., GAERS, WAG/Rij, and monogenic mutants), sample preparation approaches (gel-based urea/thiourea buffers, NP-40 extraction, and urea-based peptide workflows), and analytical platforms (2-DE/2D-DIGE, LC–MS/MS with DDA/DIA, and MALDI imaging). Together, these methodological differences affect proteome coverage and limit cross-study comparability. Representative studies corresponding to each model, sample preparation approach, and analytical platform are detailed in Table 1 and cited throughout Section Genetic Models of Absence Epilepsy–Section Impact of Methodological Variables on Proteomic Data Interpretation. Created in BioRender. Gunel, A. (2026) https://BioRender.com/w98v9yj (accessed on 6 February 2026).

These methodological considerations are included to clarify how differences in analytical strategy can influence the interpretation of model- and region-specific proteomic findings discussed above. The major sources of methodological heterogeneity across absence epilepsy proteomic studies are summarized in Figure 2.

Sample preparation protocols further amplify this variability. In the MALDI-imaging study by Lagarrigue et al. [17], tissues were first pulverized in liquid nitrogen and then lysed in HEPES buffer containing protease inhibitors. Following a 30-min centrifugation at 15,000× *g* the supernatant was further centrifuged at 105,000× *g* for 1 h. This protocol helps eliminate nucleic acid contamination and remove lipids which could otherwise interfere with proteome detection.

Moreover, with Harutyunyan et al. [11], brain tissues from cortico–thalamo–cortical circuits were also pulverized in liquid nitrogen and homogenized in Tris buffer containing 4% SDS. The homogenates were then centrifuged at 16,000× *g* for 10 min. This method facilitates efficient protein extraction, particularly from membrane-associated proteins, and ensures sufficient solubilization for downstream MS/MS analysis. The use of 4% SDS by Harutyunyan et al. [11] contrasts with the HEPES buffer used by Lagarrigue et al. [17], representing another layer of methodological variation that can significantly impact protein solubility and the final proteome coverage. In the shotgun proteomic study by Harutyunyan et al. [11] Data-Independent Acquisition (DIA) mass spectrometry was employed, which differs from the conventional Data-Dependent Acquisition (DDA) method. DIA offers advantages such as improved reproducibility and a broader dynamic range, but also presents challenges, including the need for comprehensive spectral libraries and complex data analysis [45].

Although discovery-based gel-free methods generate extensive datasets, the application of Targeted Proteomics (e.g., Selected Reaction Monitoring [SRM] and Parallel Reaction Monitoring [PRM] [46,47] remains a significant gap in absence epilepsy research. Unlike shotgun approaches, these targeted strategies allow for the precise quantification and validation of specific biomarker candidates (e.g., specific ion channel isoforms or low-abundance signaling proteins) with superior sensitivity and reproducibility. Integrating such targeted strategies—particularly PRM for high-resolution validation—in future studies will be important for validating findings derived from global proteomic profiling.

Lastly, the integration of Trapped Ion Mobility Spectrometry (TIMS) coupled with Parallel Accumulation–Serial Fragmentation (PASEF) [48] represents an emerging analytical approach. Unlike standard MS methods that separate ions solely based on mass-to-charge ratio, TIMS adds a fourth dimension of separation based on ion mobility. This allows for the measurement of Collisional Cross Section (CCS) values, effectively resolving isomers and revealing conformational properties of proteins. Given that absence epilepsy is fundamentally a channelopathy involving complex ion channel gating mechanisms, the ability of TIMS to distinguish between different protein conformers or post-translational modification (PTM) isomers could uncover structural subtleties in the thalamocortical circuitry that traditional shotgun proteomics misses.

##### Overview of Proteomic Methodologies Used in Absence Epilepsy Studies

Proteomic studies conducted in animal models of absence epilepsy have employed a range of analytical strategies, including gel-based, gel-free, and imaging-based approaches. These methodologies differ substantially in terms of sample preparation, analytical depth, spatial resolution, and protein class coverage, and therefore provide complementary—but not directly interchangeable—views of the proteome.

Two-dimensional gel electrophoresis was among the earliest proteomic techniques applied in absence epilepsy research. Since its introduction by Klose and O’Farrel in the 1970s [32,33], 2-DE has been widely used to investigate region-specific protein expression changes in both genetic and pharmacological models. 2-DIGE represents a methodological refinement of conventional 2-DE, enabling multiple samples to be analyzed within a single gel through fluorescent labeling, thereby reducing gel-to-gel variability and improving quantitative reliability [34]. Both approaches rely on isoelectric focusing in the first dimension and SDS–PAGE in the second dimension.

Across gel-based studies discussed in this review, sample preparation protocols typically involved urea/thiourea-based solubilization buffers supplemented with detergents such as 3-((3-cholamidopropyl) dimethylammonio)-1-propanesulfonate (CHAPS), ampholytes, reducing agents, and protease inhibitor cocktails, although deviations from this standard workflow have been reported. Variations in detergent composition and extraction buffers influence protein solubilization efficiency, particularly for membrane-associated and low-abundance proteins and may contribute to differences in detected proteomes across studies.

In contrast to gel-based workflows, gel-free proteomic approaches are generally used to achieve deeper and more comprehensive proteome coverage through peptide-based mass spectrometry analysis. Shotgun proteomics, which involves enzymatic digestion of proteins followed by LC-MS/MS analysis, has been applied more recently in absence epilepsy research, mainly within the context of multi-omics studies. These approaches improve the detection of hydrophobic and membrane-associated proteins but typically rely on bulk tissue homogenization, resulting in a loss of spatial information. Spatial proteomics, most notably Matrix-Assisted Laser Desorption/Ionization Imaging Mass Spectrometry (MALDI-IMS), addresses this limitation by preserving tissue architecture and enabling the visualization of molecular distributions directly within histological sections. This aspect becomes particularly relevant in absence epilepsy, where circuit-level alterations within the thalamocortical network are thought to play a central role in seizure generation.

In addition, methodological diversity extends to the mass spectrometry platforms and acquisition strategies employed across studies. Differences in dynamic range, sensitivity, spatial resolution, and protein class coverage influence which subsets of the proteome can be detected using gel-based, gel-free, and imaging-based workflows. Consequently, the choice of proteomic methodology should be guided by the specific biological question being addressed rather than considered interchangeable across studies.

Emerging analytical strategies, such as Trapped Ion Mobility Spectrometry (TIMS) coupled with Parallel Accumulation–Serial Fragmentation (PASEF), further expand proteomic resolution by introducing ion mobility as an additional dimension of separation. These developments offer the potential to resolve isomeric species and conformational variants, which may be particularly relevant for ion channel-centered mechanisms underlying absence epilepsy.

### 3.2. Current Challenges and Methodological Considerations in Absence Epilepsy Proteomics

Since the term proteome was first introduced to the literature, the aforementioned procedures have been applied in gel-based and shotgun proteomic methodologies to study absence epilepsy, one of the inherited epileptic syndromes of childhood. Detergents used in sample preparation in gel-based approaches are non-ionic detergents because they do not disrupt the isoelectric focusing process, which is the first step of the two-dimensional electrophoresis method and is particularly sensitive to different salts and chemicals. Similarly, in shotgun proteomics, the choice of lysis buffer (e.g., SDS vs. chaotropic buffers), digestion protocols, and mass spectrometric acquisition methods (DDA vs. DIA) adds further layers of variability.

Gel-free LC-MS/MS covers membrane proteins; gel-based methods struggle with low-abundance proteins that are central to disease mechanisms. MALDI imaging gives region-specific data, but context matters. Overall, these approaches should be viewed as complementary, with the methodological choice guided by the specific research question.

Although the combined application of 2-DE and shotgun proteomics poses practical challenges for clinical translation due to increased workload and time requirements, this dual strategy remains valuable for comprehensive proteome analysis. As noted by Westermeier, “there’s life in the old dog yet” [49], reflecting the continued relevance of well-established proteomic methods. At the same time, their effective use increasingly depends on implementation within more standardized analytical frameworks to improve reproducibility.

Future progress in absence epilepsy proteomics depends on the development of standardized protocols across key experimental steps to improve cross-study comparability. In particular, greater consistency is required in the following: (1) the selection of animal models, brain region dissection strategies, and age-matched controls, in line with the methodological standardization initiatives proposed by the AES/ILAE Joint Translational Task Force [50]; (2) the implementation of optimized and reproducible sample preparation workflows tailored to brain tissue; (3) the critical selection of mass spectrometric acquisition strategies (e.g., DDA vs. DIA) according to the specific research question, while accounting for the strengths and limitations of each approach—DIA offers advantages in reproducibility and quantitative precision but requires complex spectral libraries and data analysis, whereas DDA remains a robust and accessible discovery tool—and crucially; (4) addressing the significant sex disparity in preclinical models. Given that absence epilepsy has a higher incidence in females [51] in the clinical population, the predominant use of male animals in experimental studies represents a major translational gap. Future studies must incorporate both sexes to identify potential sex-specific molecular mechanisms and ensure the broader relevance of proteomic findings.

In addition to standardized wet-lab protocols, effective interpretation of proteomic data increasingly relies on the development and application of robust bioinformatic pipelines for data analysis and integration. The heterogeneity inherent to proteomic datasets requires advanced computational tools for cross-study comparison, pathway analysis, and integration with other omics layers (e.g., transcriptomics and metabolomics) to support a systems-level understanding of absence epilepsy. In this context, the establishment of centralized and curated databases for epilepsy-related proteomic data represents an important infrastructural component for the field.

Collectively, these insights contribute to the identification of potential therapeutic targets and candidate biomarkers. Validation of proteomic findings derived from animal models depends on their translational assessment in human studies, including analyses of cerebrospinal fluid or blood-based biomarkers, as well as functional characterization in disease-relevant cellular systems. Within this framework, methodologically rigorous and integrative preclinical proteomic approaches provide a critical foundation for addressing the translational challenges associated with absence epilepsy.

#### Future Perspectives: Data-Driven and Multivariate Approaches in Absence Epilepsy Research

Proteomic investigations in absence epilepsy typically reveal subtle molecular alterations, involvement of distributed networks, and an absence of single dominant biomarkers capable of accounting for seizure generation. Such characteristics complicate the use of conventional univariate statistical analyses, particularly in studies with limited sample sizes. To date, applications of artificial intelligence and machine learning in proteomic research have primarily focused on heterogeneous epilepsy cohorts rather than absence epilepsy specifically [52,53].

In these broader epilepsy populations, multivariate analytical strategies such as Random Forest and sparse Partial Least Squares Discriminant Analysis have been used to prioritize coordinated protein patterns and consensus biomarker panels associated with seizure activity [52,53]. Across these studies, enrichment of immune-related and neuroinflammatory pathways emerges as a recurring finding, suggesting that seizure-associated proteomic alterations are more accurately captured at the level of coordinated protein networks rather than individual markers.

Although comparable machine learning-based proteomic analyses have not yet been conducted specifically in absence epilepsy, methodological insights from related studies offer a useful reference for the field. In the context of diffuse thalamocortical network dysfunction and modest effect sizes commonly reported in absence epilepsy, multivariate and data-driven analytical strategies are well suited to detect subtle yet biologically coherent molecular patterns. These approaches are best considered complementary tools for hypothesis generation and require subsequent biological validation in absence epilepsy-specific cohorts.

Beyond proteomic profiling, data-driven analytical approaches have increasingly been applied to other molecular and imaging modalities in epilepsy research, providing methodological frameworks that may inform future studies in absence epilepsy. In transcriptomic analyses of focal epilepsies, particularly temporal lobe epilepsy, interpretable deep learning models have achieved high diagnostic performance while identifying biologically relevant genes, including DEPDC5 and STXBP1, through explainable architectures such as SHAP (SHapley Additive exPlanations) and Kolmogorov–Arnold Networks (KAN) [54]. Although these findings originate from focal epilepsy cohorts, they illustrate how integrating model interpretability with molecular feature selection can support biologically meaningful inference. These examples are discussed solely to illustrate methodological and analytical frameworks and do not imply disease specificity for absence epilepsy.

Machine learning-based analyses of plasma metabolic fingerprints have been applied to epilepsy screening in clinically heterogeneous cohorts, allowing high-throughput classification and reduced diagnostic turnaround times [55]. In parallel, multivariate pattern analysis of multimodal neuroimaging data has shown that Support Vector Machine-based classifiers can distinguish pediatric epilepsy patients from healthy controls by integrating structural and functional MRI features, particularly within thalamocortical networks [56]. Although these studies do not specifically address absence epilepsy, they demonstrate how multivariate and interpretable computational frameworks can detect subtle and spatially distributed disease signatures that are difficult to resolve using conventional analytical approaches. In this context, the application of interpretable, multi-modal computational pipelines to absence epilepsy cohorts represents a plausible translational direction for integrating molecular and imaging data into subtype-specific diagnostic and therapeutic strategies.

## 4. Conclusions

One practical implication of the findings reviewed here is the clear need for shared methodological standards that can improve reproducibility and comparability across studies. In this context, the continued underrepresentation of female animals in preclinical proteomic research remains a significant limitation, as it restricts the extent to which experimental results can be considered representative of the clinical phenotype and should therefore be addressed in future work.

At the same time, proteomic data alone are unlikely to capture the full complexity of disease mechanisms. Meaningful progress will depend on the integration of proteomics with additional omics layers, such as lipidomics, glycomics, and metabolomics, within a systems-level framework. Addressing existing methodological constraints alongside such integrative strategies will be essential for improving consistency across studies and for strengthening the translational relevance of future research.

## Figures and Tables

**Table 1:** Summary of proteomic studies conducted in absence epilepsy.

Study	Model	Brain Region	Sample Preparation	Proteomic Method	Main Findings
Danış et al., 2011 [9]	Genetic Absence Epilepsy Rats from Strasbourg (GAERS)	Cortex; Thalamus; Hippocampus	2-DE buffer	2-DE + nanoLC-MS/MS (DDA mode)	MBP, MIF, 14-3-3 alterations
Yuce-Dursun et al., 2014 [10]	GAERS	Cortex membrane fraction	2-DE	2-DE + MALDI	↑ 14-3-3, ↓ G-protein beta-1
Harutyunyan et al., 2022 [11]	GAERS	S1 cortex; Thalamus	4% SDS	DIA-MS	Synaptic & oxidative ↑; lysine catabolism ↓
Györffy et al., 2014 [12]	Wistar Albino Glaxo rats from Rijswijk (WAG/Rij), (WAG/Rij) (+LPS)	Cortex; Thalamus	2D-DIGE	LC-MS/MS (DDA mode)	Immune activation (NF-κB pathway)
Gürol et al., 2015 [13]	WAG/Rij	Frontal Cortex; Thalamus	NP-40 + 2-DE	2-DE + MALDI-TOF	Vesicle transport, cytoskeleton changes
Sahin et al., 2018 [14]	WAG/Rij	Cortex; Thalamus	2-DE	MALDI-TOF/TOF	↓ ERP57
Ryu et al., 2008 [15]	Stargazer	Thalamus	DIGE buffer	2D-DIGE + MALDI	↓ Metabolic enzymes, oxidative stress proteins
Ryu et al., 2007 [16]	GBL-treated mouse	Thalamus	DIGE buffer	2D-DIGE + MALDI	↓ Cytoskeleton proteins, ↓ neuroprotection, CRMP phosphorylation
Lagarrigue et al., 2012 [17]	BS/Orl vs. BR/Orl	Whole section	HEPES buffer	MALDI-IMS	Synapsin-I differences

GBL, γ-butyrolactone; 2-DE, Two dimensional electrophoresis; SDS, Sodium dodecyl sulphate; NP-40, Nonidet P-40; DIGE; Differential gel electrophoresis; LC-MS/MS, Liquid chromatography–tandem mass spectrometry; MALDI-TOF; Matrix Assisted Laser Desorption Ionization-Time of Flight; MALDI-IMS, Matrix Assisted Laser Desorption Ionization-Imaging Mass Spectrometry; DDA, Data Dependent Acquisition; DIA-MS, Data Independent Acquisition-Mass Spectrometry; MBP, Myelin basic protein; MIF, macrophage migration inhibitory factor. ↑: Increased or upregulated, ↓: Decreased or down regulated.

## Data Availability

No new data were created or analyzed in this study. Data sharing is not applicable to this article.

## Supplementary Materials

The following supporting information can be downloaded at: https://www.mdpi.com/article/10.3390/cimb48020200/s1. Reference [57] is cited in the Supplementary Materials.
