# Supplementary material for: Proteomic Studies in Absence Epilepsy: A Systematic Review of Methodological Diversity and Implications for Data Interpretation
Source: Curr Issues Mol Biol. 2026 Feb 11;48(2):200. doi: 10.3390/cimb48020200 (PMC12939128; doi:10.3390/cimb48020200)
Supplement: Supplementary file 1 [file cimb-48-00200-s001.zip › cimb-4133738-supplementary.pdf]

# PRISMA 2020 Checklist

| Section and Topic | Item # | Checklist item | Location where item is reported |
|---|---|---|---|
| **TITLE** | | | |
| Title | 1 | Identify the report as a systematic review. | Although the review follows PRISMA guidelines, it is presented as a methodological narrative review rather than a formal systematic review. |
| **ABSTRACT** | | | |
| Abstract | 2 | The abstract provides the background, objectives, methodological focus, scope of proteomic studies included, major findings, and conclusions. The abstract summarizes the systematic search of proteomic studies in rodent models of absence epilepsy and emphasizes methodological heterogeneity and its implications for data interpretation. | Abstract |
| **INTRODUCTION** | | | |
| Rationale | 3 | The rationale is clearly described in the Introduction (pages 2–3). Previous proteomic studies in epilepsy are methodologically heterogeneous and largely focused on focal epilepsies. Absence epilepsy remains underexplored at the proteomic level, and existing studies vary substantially in model selection, sample preparation, and analytical platforms. This review addresses the need for a methodologically focused synthesis specific to absence epilepsy. | Pages 2-3 |
| Objectives | 4 | The objective of this review is to systematically identify and critically evaluate proteomic studies conducted in rodent models of absence epilepsy, with particular emphasis on methodological design, analytical workflows, and how these factors influence data interpretation and reproducibility. | |
| **METHODS** | | | |
| Eligibility criteria | 5 | Inclusion criteria were:<br>(1) Original research articles employing proteomic technologies;<br>(2) Studies conducted in established genetic or pharmacological rodent models of absence epilepsy (e.g., GAERS, WAG/Rij, Stargazer, GBL-induced models);<br>(3) Articles published in English.<br><br>Exclusion criteria included:<br>Reviews, editorials, conference papers, non-English articles, non-proteomic studies, inappropriate epilepsy models (e.g., TLE, MTLE, cortical dysplasia), and human studies. | Material and Methods |
| Information sources | 6 | A comprehensive literature search was conducted using PubMed and Scopus databases. | Material and Methods |

# PRISMA 2020 Checklist

| Section and Topic | Item # | Checklist item | Location where item is reported |
|---|---|---|---|
| Search strategy | 7 | Search terms included combinations of “absence epilepsy” AND “proteomics”, and “epilepsy” AND “proteomics”. The search covered studies published between 2002 and 2025. | Material and Methods |
| Selection process | 8 | Titles and abstracts were screened for relevance, followed by full-text evaluation based on predefined inclusion and exclusion criteria. Study selection and data extraction were conducted by a single reviewer due to the methodological and narrative focus of the review. | Material and Methods |
| Data collection process | 9 | Data were systematically extracted with a focus on animal model, brain region analyzed, sample preparation protocols, proteomic methodology, and key findings. | |
| Data items | 10a | Extracted variables included animal model type, brain region, protein extraction method, analytical platform (gel-based, gel-free, imaging-based), acquisition strategy (DDA/DIA), and reported proteomic alterations. | |
| | 10b | List and define all other variables for which data were sought (e.g. participant and intervention characteristics, funding sources). Describe any assumptions made about any missing or unclear information. | |
| Study risk of bias assessment | 11 | Formal risk-of-bias assessment tools were not applied, as the review focuses on methodological heterogeneity rather than effect estimation. | Material and Methods, Discussion |
| Effect measures | 12 | No quantitative effect measures were applied, as meta-analysis was not feasible due to methodological heterogeneity. | |
| Synthesis methods | 13a | A qualitative and critical synthesis was performed, focusing on methodological comparison rather than quantitative aggregation of results. | |
| | 13b | Describe any methods required to prepare the data for presentation or synthesis, such as handling of missing summary statistics, or data conversions. | |
| | 13c | Results were summarized using structured tables (Table 1) and schematic figures (Figure 2). | Table 1 and Figure 2 |
| | 13d | Describe any methods used to synthesize results and provide a rationale for the choice(s). If meta-analysis was performed, describe the model(s), method(s) to identify the presence and extent of statistical heterogeneity, and software package(s) used. | |
| | 13e | Describe any methods used to explore possible causes of heterogeneity among study results (e.g. subgroup analysis, meta-regression). | |
| | 13f | Describe any sensitivity analyses conducted to assess robustness of the synthesized results. | |
| Reporting bias assessment | 14 | Formal assessment of reporting bias was not performed. Potential publication bias is acknowledged as a limitation. | |
| Certainty assessment | 15 | Certainty of evidence was not formally graded. The review emphasizes methodological rigor and reproducibility rather than evidence grading. | |
| **RESULTS** | | | |
| Study selection | 16a | The PRISMA 2020 flow diagram (Figure 1) summarizes the identification, screening, eligibility assessment, and inclusion of studies. A total of 10 studies were included. | Material and Methds, Figure 1 |
| | 16b | Cite studies that might appear to meet the inclusion criteria, but which were excluded, and explain why they were excluded. | |

# PRISMA 2020 Checklist

| Section and Topic | Item # | Checklist item | Location where item is reported |
|---|---|---|---|
| Study characteristics | 17 | Key characteristics of included studies are summarized in Table 1, detailing animal models, brain regions, sample preparation methods, proteomic platforms, and major findings. | Table 1 |
| Risk of bias in studies | 18 | Risk of bias was addressed narratively through discussion of methodological limitations rather than formal scoring. | |
| Results of individual studies | 19 | Individual study findings are described in detail in Sections 3.1.2.1–3.1.2.4. | Sections 3.1.2.1–3.1.2.4 |
| Results of syntheses | 20a | Results are synthesized by model type and proteomic methodology, highlighting convergent and divergent findings across studies. | |
| | 20b | Present results of all statistical syntheses conducted. If meta-analysis was done, present for each the summary estimate and its precision (e.g. confidence/credible interval) and measures of statistical heterogeneity. If comparing groups, describe the direction of the effect. | |
| | 20c | Present results of all investigations of possible causes of heterogeneity among study results. | |
| | 20d | Present results of all sensitivity analyses conducted to assess the robustness of the synthesized results. | |
| Reporting biases | 21 | Reporting bias is discussed qualitatively as a limitation due to the small number of studies and variability in reporting standards. | |
| Certainty of evidence | 22 | The certainty of evidence is discussed narratively, emphasizing methodological diversity and the need for standardized protocols. | |
| **DISCUSSION** | | | |
| Discussion | 23a | The Discussion contextualizes findings within existing epilepsy and proteomics literature, focusing on methodological implications and translational relevance. | |
| | 23b | Limitations include methodological heterogeneity, lack of standardized protocols, small number of studies, and absence of formal meta-analysis. | |
| | 23c | Discuss any limitations of the review processes used. | |
| | 23d | The review highlights the need for standardized proteomic workflows and integrative multi-omics approaches in absence epilepsy research. | |
| **OTHER INFORMATION** | | | |
| Registration and protocol | 24a | This review was not registered in PROSPERO or a similar database, and no formal protocol was prepared. | This review was not registered in PROSPERO or a similar database. |
| | 24b | Indicate where the review protocol can be accessed, or state that a protocol was not prepared. | |
| | 24c | Describe and explain any amendments to information provided at registration or in the protocol. | |
| Support | 25 | No external funding was received. | |
| Competing interests | 26 | The author declares no competing interests. | |

# PRISMA 2020 Checklist

| Section and Topic | Item # | Checklist item | Location where item is reported |
|---|---|---|---|
| Availability of data, code and other materials | 27 | No new data were generated or analyzed in this review. | |

*From:* Page MJ, McKenzie JE, Bossuyt PM, Boutron I, Hoffmann TC, Mulrow CD, et al. The PRISMA 2020 statement: an updated guideline for reporting systematic reviews. BMJ 2021;372:n71. doi: 10.1136/bmj.n71. This work is licensed under CC BY 4.0. To view a copy of this license, visit https://creativecommons.org/licenses/by/4.0/
